# Roxadustat for the treatment of anemia in patients with chronic kidney diseases: a meta-analysis

**DOI:** 10.18632/aging.203143

**Published:** 2021-06-11

**Authors:** Li Zhang, Jie Hou, Jia Li, Sen-Sen Su, Shuai Xue

**Affiliations:** 1 Department of Nephrology, The First Hospital of Jilin University, Jilin Province, China; 2 Department of Thyroid Surgery, The First Hospital of Jilin University, Jilin Province, China

**Keywords:** roxadustat, chronic kidney disease, anemia, meta-analysis

## Abstract

Background: Anemia is a common complication of chronic kidney disease (CKD). Treating renal anemia with erythropoiesis-stimulating agents (ESAs) or erythropoietin analogs is effective but has side effects. Therefore, we performed a meta-analysis to assess the efficacy and safety of roxadustat in treating CKD-induced anemia.

Methods: We searched publications online and conducted a meta-analysis and calculated relative risks with 95% confidence intervals (CIs) for dichotomous data and mean differences (MD) with 95% CIs for continuous data.

Results: Of 110 articles, nine were included that contained 12 data sets and 11 randomized control trials on roxadustat. In the non-dialysis-dependent (NDD) high-dose/low-dose subgroups, the change in hemoglobin (Hb) levels was significantly higher in the roxadustat group than in the placebo group (*P*<0.0001, *P*=0.001, respectively). The Hb response rate of the roxadustat is higher in the NDD subgroup than in the placebo group (*P*<0.00001, MD=6.92, 95% CI: 4.03, 11.89). However, in the dialysis-dependent subgroup, there was no significant difference in the change in Hb levels or the Hb response rate between the roxadustat and ESA groups. There was no change in the mortality in the roxadustat group compared to that in the placebo/ESA group. Hyperkalemia may be a side effect of roxadustat.

Conclusions: Roxadustat elevated the serum Hb levels in a manner similar to that observed for ESAs. Roxadustat raised the Hb levels more significantly than the placebo and showed a higher Hb response rate than the placebo group in NDD patients. Roxadustat is a safe and effective drug for anemia in CKD patients.

## INTRODUCTION

The prevalence of chronic kidney disease (CKD) is increasing globally [1]. Anemia is one of the most common complications of CKD, with nearly 50% of patients in III‒V stage CKD developing anemia [2]. The number of patients suffering from anemia is higher in the dialysis-dependent (DD) patient population [3]. The treatment of anemia in CKD is mainly performed clinically using erythropoiesis-stimulating agents (ESAs) or erythropoietin (EPO) analogs [4]. However, there are many side effects of this treatment, including cardiovascular events, stroke, hypertension, hypersensitivity reactions, thrombotic risks, susceptibility to infectious diseases, increased cancer risk, and even a higher risk of death [5–8]. Furthermore, there is a risk of ~10% of hemodialysis patients developing resistance to ESAs [4]. Therefore, a safer and more effective treatment is urgently needed for anemia in CKD patients.

Hypoxia-inducible factors (HIFs) regulate the expression of genes in response to hypoxia. These genes include those required for erythropoiesis and iron metabolism. HIF-prolyl hydroxylases (HIF-PHs) degrade HIF-α at normal oxygen concentrations. At low oxygen levels, HIF-PH activity decreases, which activates transcriptional programs resulting in the promotion of erythropoiesis [9, 10]. HIF-PH inhibitors (HIF-PHIs) inhibit the degradation of HIF-α, which then translocates into the nucleus with HIF-β to activate the transcription of genes related to erythropoiesis [10]. HIF-PHI therapy is currently the most promising drug treatment for anemia in CKD. Roxadustat is an oral HIF-PHI that is also known as FG-4592. Many phase II and phase III roxadustat clinical trials have reported that roxadustat can stimulate endogenous EPO and inhibit hepcidin expression, which improves iron absorption and utilization. Thus, roxadustat can elevate the Hb levels in anemic patients through this iron-dependent mechanism. However, there have been no studies on the safety and efficacy of roxadustat in CKD patients in comparison to ESAs [11]. Therefore, we performed this meta-analysis of clinical data on roxadustat to assess the efficacy and safety of its use in anemic CKD patients.

## RESULTS

### Search results

**Figure 1:**
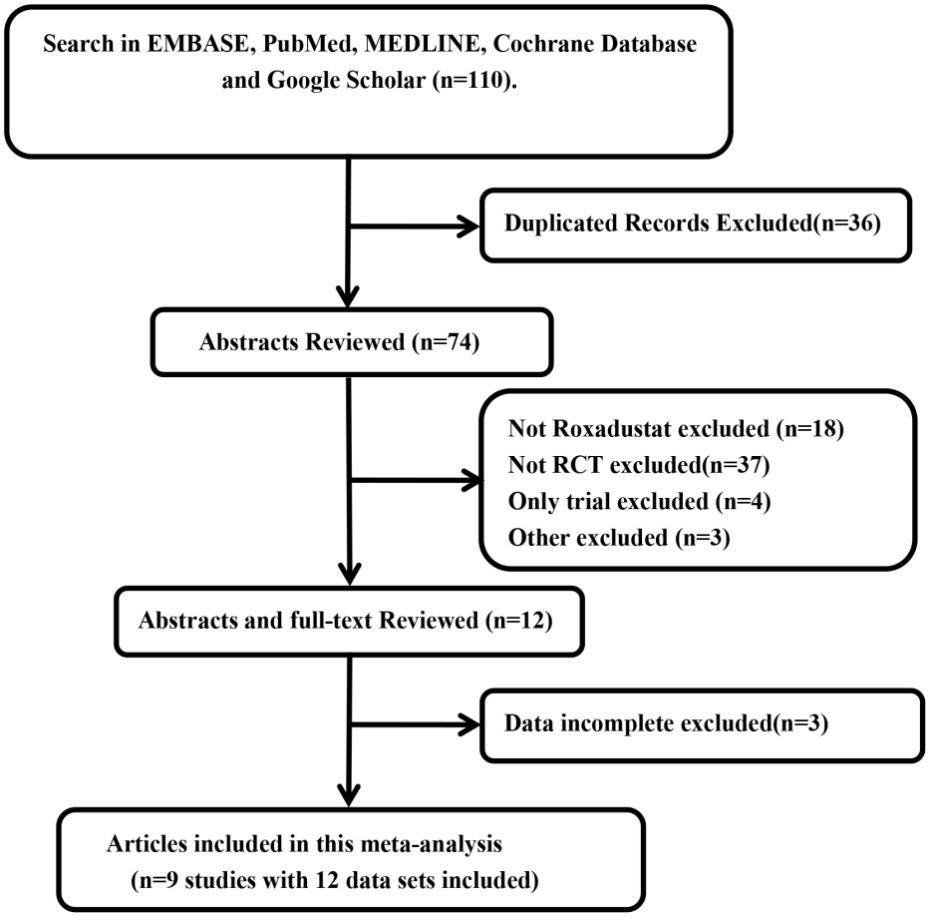
Flow gram of search and selection of studies.

We identified 110 articles by searching EMBASE, PubMed, MEDLINE, Cochrane Database, and Google Scholar without any limitations on language. One hundred and one of these articles were either duplicated, did not include roxadustat, were not randomized controlled trials (RCTs), were only clinical trials, or contained incomplete data and were therefore excluded. Finally, nine articles [12–20] that included 12 data sets and 11 RCTs (Figure 1) were used to perform the meta-analysis.

### Study characteristics

In this meta-analysis, Provenzano's study included two different trial methods (6-week and 19-week roxadustat treatments). Therefore, we considered this study to consist of two data sets—part 1 (P1) and part 2 (P2)—and one RCT. Both Chen's and Esposito's studies [13, 14] included two different RCTs, which we named P1 (non-dialysis-dependent, NDD) and P2 (dialysis-dependent, DD), respectively. Among the 11 RCTs, there were five phase II clinical trials and six phase III clinical trials; six trials examined NDD-CKD and five DD-CKD. In the NDD studies, the control was a placebo, whereas in the DD studies, the control was epoetin alfa (EA), an ESA. There were six open-label trials [13, 15, 17, 19, 20], of which one was a phase 2 clinical trial [16] that included an initial 8-week, double-blind, placebo-controlled phase and an 18-week, open-label phase; only the initial phase was included in this meta-analysis. There were five randomized double-blind, placebo-controlled trials [14–16, 18, 20] and one randomized single-blind, placebo-controlled trial [12]. There were three conference abstracts from the 2019 Kidney Week from the American Society of Nephrology [18–20] that reported studies on 4024 cases and 3372 controls. There was no significant difference in the baseline characteristics of age, sex, estimated glomerular filtration rate (eGFR), hemoglobin (Hb), percent transferrin saturation (TSAT%), ferritin, and hepcidin levels (Supplementary Table 1) between the roxadustat and control groups.

### Study quality

**Figure 2:**
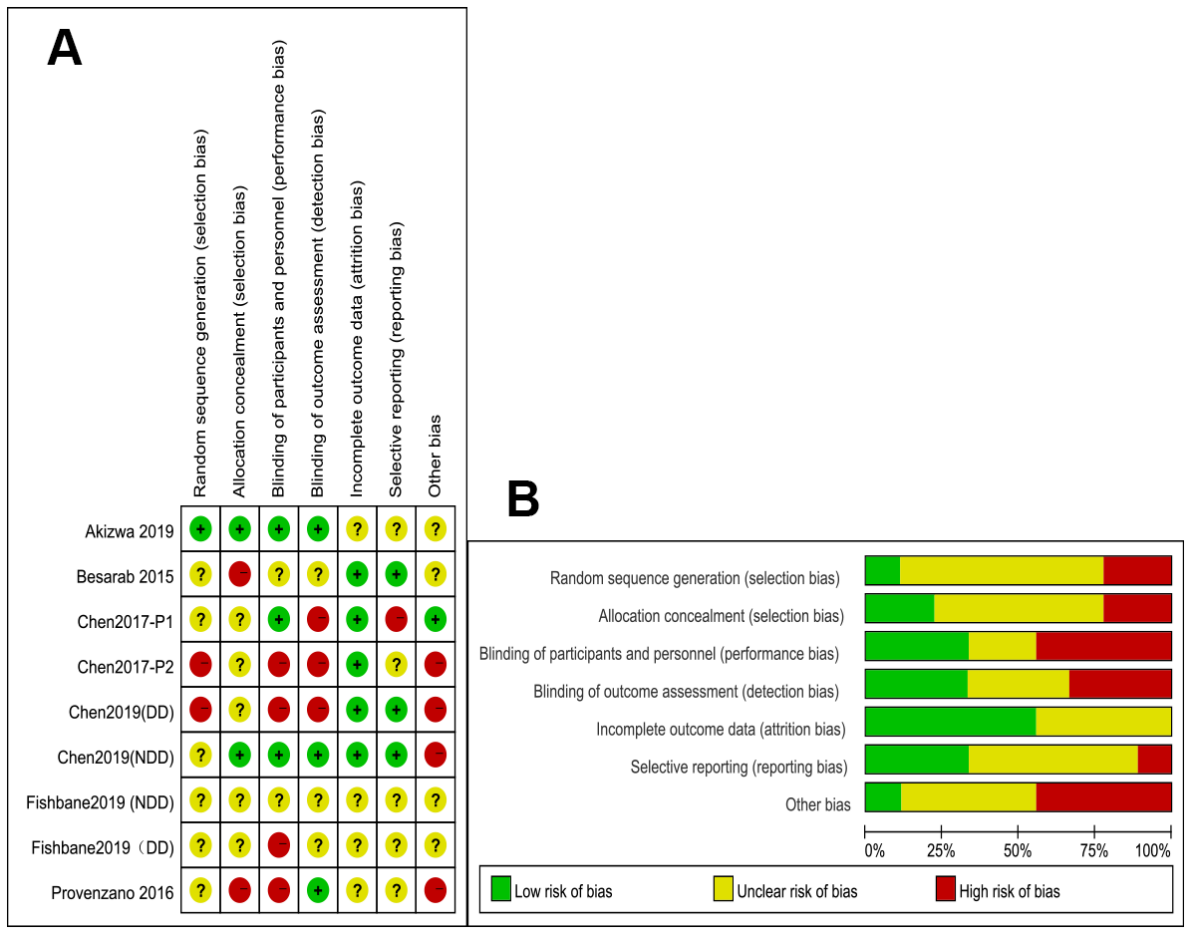
(**A**) Summary of the quality assessment of the included studies; (**B**) Quality assessment graph.

The risk of bias assessment summary is shown in Figure 2. Only one RCT [15] recorded how the randomization process was performed. The description of how the allocation concealment was performed was unclear. There were five open-label studies that may have introduced a performance bias [13, 15, 17, 19, 20]. However, because the primary outcomes were detected by laboratory methods, the results of this meta-analysis are less likely to be influenced by the open-label study design. The results of four RCTs were reported as conference abstracts or oral presentations [18–20], because of which there was not enough information to judge the bias of these studies. As we included <10 studies in our meta-analysis, we could not assess the publication bias using a funnel plot.

### Primary outcomes: Hb-related comparisons

### Change in Hb levels from baseline

**Figure 3:**
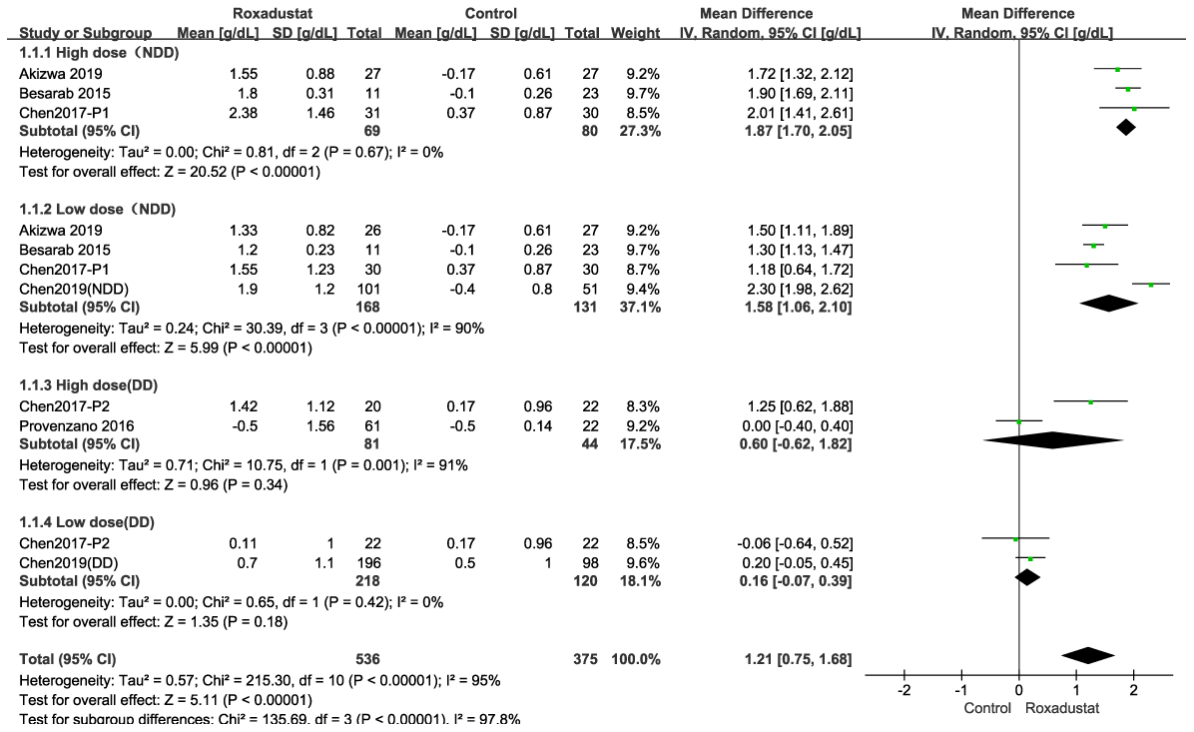
Roxadustat effect on Hb change. Forest plots for the subgroup of (**A**) High dose in NDD (**B**) Low dose in NDD. (**C**) High dose in DD. (**D**) Low dose in DD. In the NDD studies the control was placebo, and in the DD studies the control was EA or ESA.

Because the meta-analysis could be affected by the roxadustat dosage in the six articles [12–17] and whether the patients got dialysis, we performed four subgroup analyses depending on whether the patient received a high/low dose of roxadustat or whether the patient had NDD/DD-CKD (Figure 3). In the high-dose NDD subgroup, there was no significant heterogeneity, and the Hb change was significantly higher in the roxadustat group than in the placebo group (*P*<0.0001, mean difference [MD]=1.87 [95% confidence interval (CI): 1.70, 2.05], Figure 3A). In the low-dose NDD subgroup, there was significant heterogeneity (I^2^=98%), and the Hb level change was significantly higher in the roxadustat group (*P*=0.001, MD=1.29 [95% CI: 0.50, 2.09], Figure 3B). There was no significant difference in the Hb level changes between the roxadustat and the ESA groups among the high- and low-dose DD-CKD subgroups (Figure 3C, 3D).

### Hb response rate

**Figure 4:**
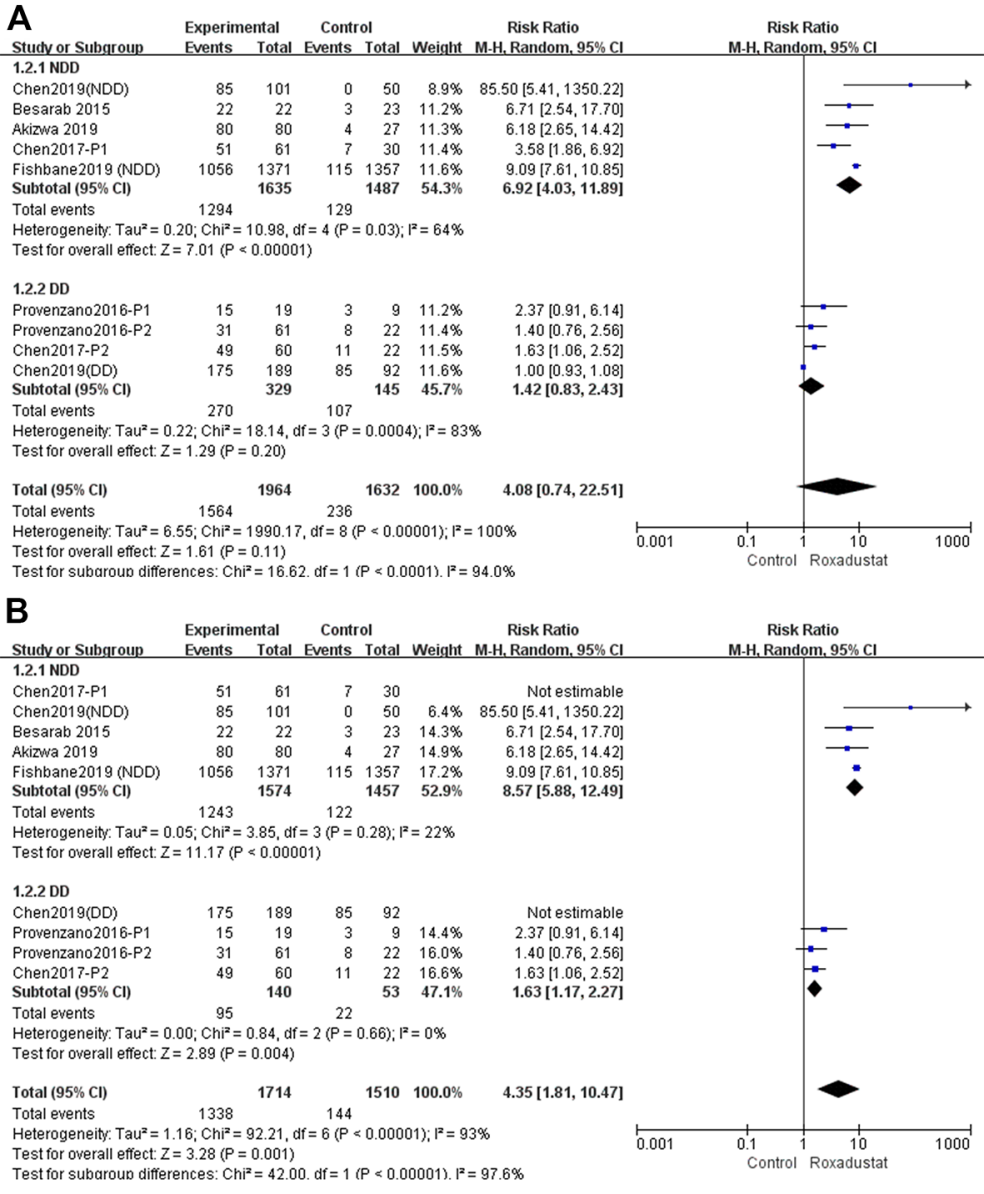
**Roxadustat effect on Hb response rate of NDD and DD subgroups.** (**A**) All studies; (**B**) Sensitive analysis.

The Hb response rate was defined as the proportion of patients whose Hb level: i) increase from the baseline was no less than 1 g/dL; or ii) was maintained at no less than 0.5 g/dL at baseline; or iii) was maintained at no less than 10.0 g/dL [14–20]. A subgroup analysis was performed according to whether the patients were diagnosed with NDD- or DD-CKD. In the NDD subgroup, the Hb response rate was significantly higher in the roxadustat group than in the placebo group (*P*<0.00001, MD=6.92, 95% CI: 4.03, 11.89) with significant heterogeneity (I^2^=64%). In the DD subgroup, there was no difference between the roxadustat and the ESA groups (*P*=0.20); however, there was significant heterogeneity (Figure 4A).

We performed a sensitivity analysis as shown in Figure 4B. After Chen 2017 P1 and Chen 2019 (DD) were excluded from the NDD and DD subgroups, one by one, the Hb response rate of the roxadustat group was significantly higher than in the control group, without significant heterogeneity.

### Mortality comparison between roxadustat and control groups

**Figure 5:**
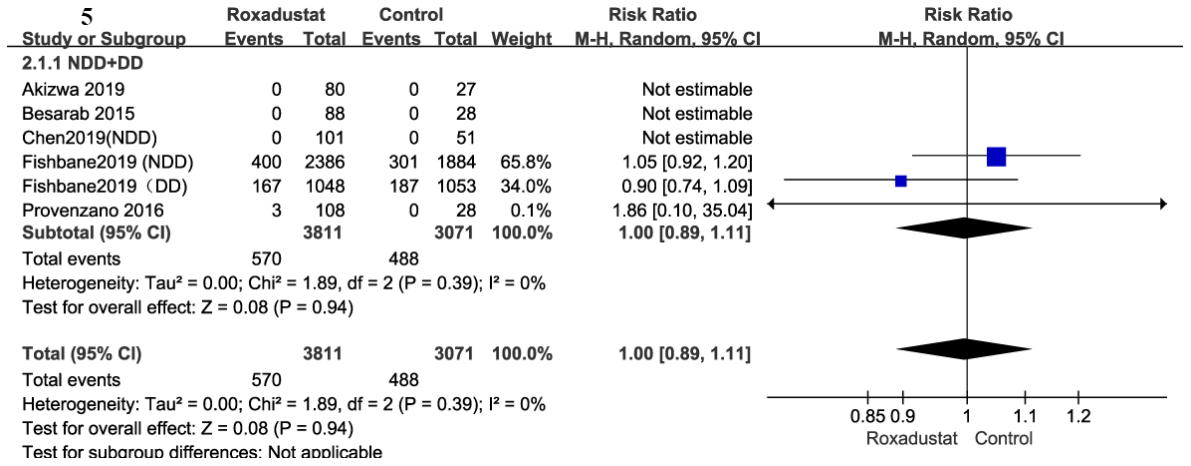
Roxadustat effect on mortality.

A total of 6882 participants were included to determine mortality in the six trials [14, 15, 17, 18, 20, 21] in this meta-analysis. Compared to the placebo/ESA groups, there was no significant difference in the mortality of the roxadustat group (*P*=0.94), and there was no significant heterogeneity (Figure 5).

### Change from baseline in iron utilization parameters

### Serum hepcidin

**Figure 6:**
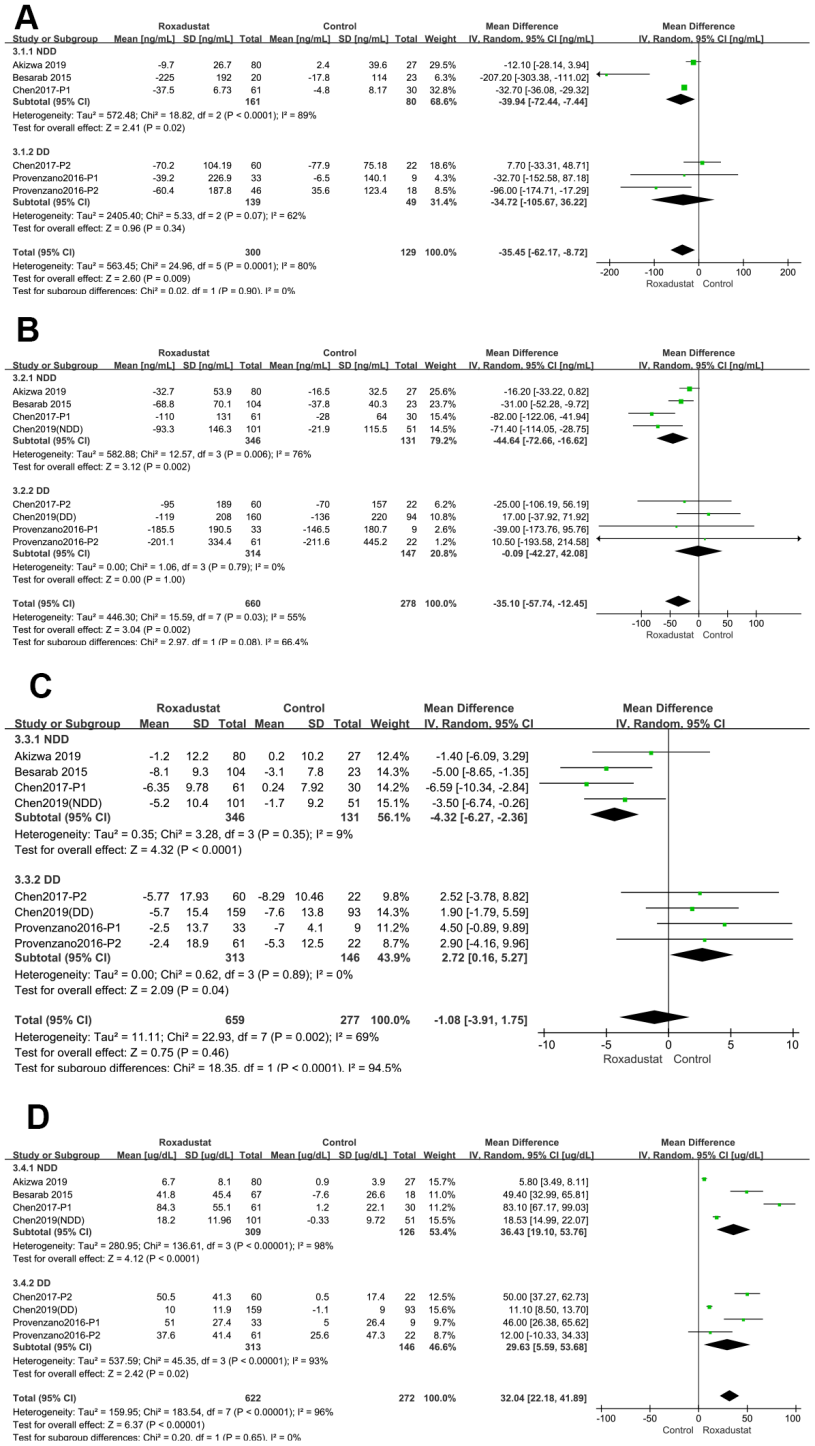
**Roxadustat effect on iron use parameters.** Forest plots of (**A**) Serum hepcidin (**B**) Serum ferritin; (**C**) ΔTSAT%; (**D**) ΔTIBC.

Serum hepcidin levels were examined in the four studies in this meta-analysis [14–17], which included six clinical trials. The pooled *P*-value of the change in serum hepcidin levels was 0.009, with significant heterogeneity (I^2^=80%). The subgroup analysis was performed similarly to the previous analyses. In the NDD subgroup, there was a significant decrease in serum hepcidin levels in the roxadustat group compared to those in the placebo group (*P*=0.02, MD=-39.94, 95% CI: -72.44, -7.44) with significant heterogeneity (I^2^=89%). There was no significant difference in the serum hepcidin levels in the DD subgroup and no significant heterogeneity compared to the ESA group (Figure 6A).

### Serum ferritin

We compared serum ferritin levels in the six studies in this meta-analysis [14–19], which included eight clinical trials. The pooled *P*-value of the change in serum ferritin levels was 0.002, with significant heterogeneity (I^2^=55%). The results of the subgroup analysis revealed a significant decrease in the serum ferritin levels in the roxadustat compared to the placebo group (*P*=0.002, MD = -44.64, 95% CI: -72.66, -16.62), with significant heterogeneity (I^2^=76%) in the NDD subgroup. There was no significant difference in the serum ferritin levels of the DD and ESA subgroups and no significant heterogeneity (Figure 6B).

### Percent change in transferrin saturation (ΔTSAT%)

ΔTSAT% was compared in NDD-CKD patients from four clinical trials [14, 16–18]. The ΔTSAT% of the roxadustat group was significantly lower than that of the placebo/ESA group (*P*<0.0001, MD = -4.32, 95% CI: -6.27, -2.36) with no significant heterogeneity. A similar result was observed in the DD-CKD subgroup [15, 16, 19] (*P*=0.04, MD=2.27, 95% CI: 0.16, 5.27); however, the ΔTSAT% of the roxadustat group was significantly lower than that of the placebo/ESA group (Figure 6C).

### Change in total iron-binding capacity (ΔTIBC)

We compared TIBC values from six studies [14–19], which included eight clinical trials. The pooled *P*-value of the ΔTIBC was <0.00001 with significant heterogeneity (I^2^=96%). In the subgroup analysis, both two subgroups showed significant heterogeneity, and the TIBC was significantly higher in the roxadustat group than in the placebo/ESA group (*P*<0.0001, *P*=0.02, respectively) (Figure 6D).

### Adverse events

### Treatment-emergent adverse events (TEAEs) and Serious AEs (SAEs)

**Figure 7:**
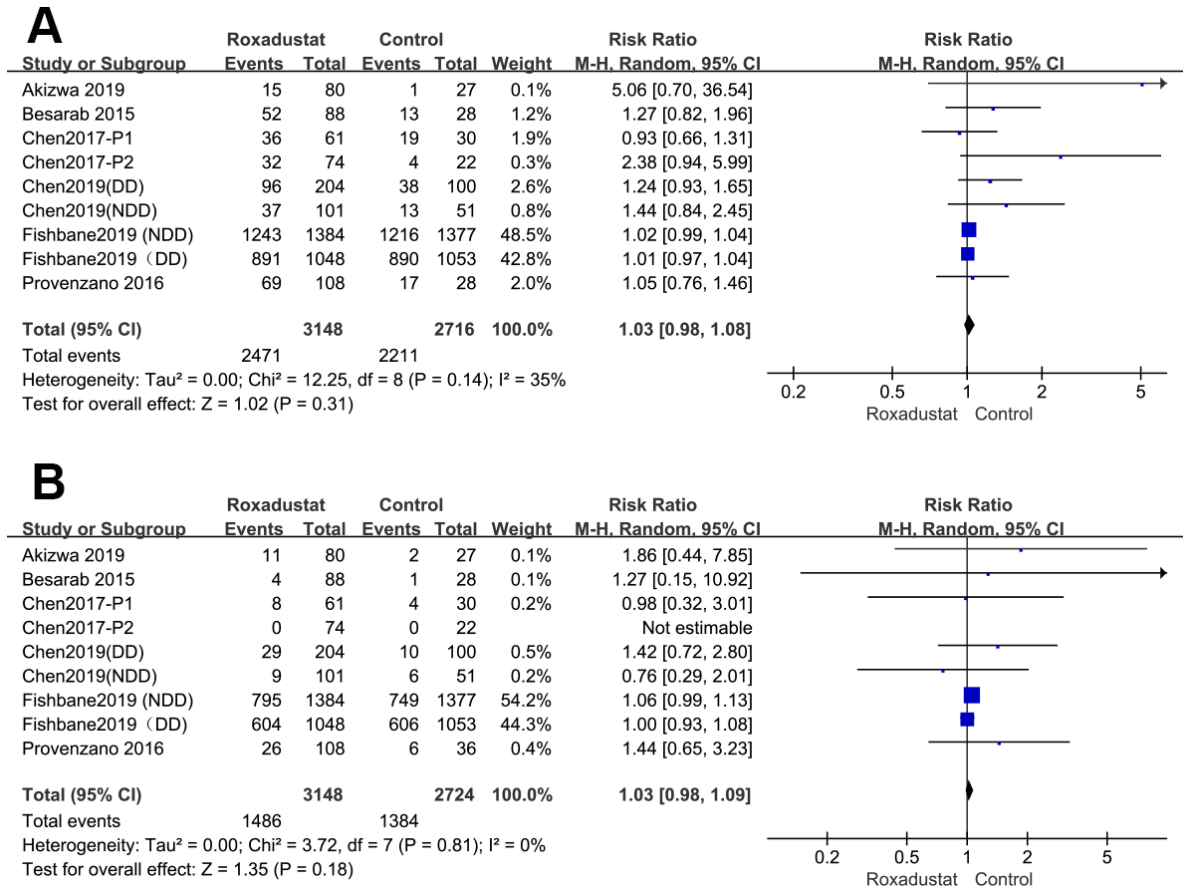
Roxadustat effect on TEAE (**A**) and SAE (**B**).

All eight articles [14–21] reported TEAEs and SAEs. There was no significant difference between the roxadustat and the placebo/ESA groups in the TEAEs (*P*=0.31, heterogeneity I^2^=35%) and the SAEs (*P*=0.18, heterogeneity I^2^=0%) (Figure 7A, 7B).

### Common AEs

**Figure 8:**
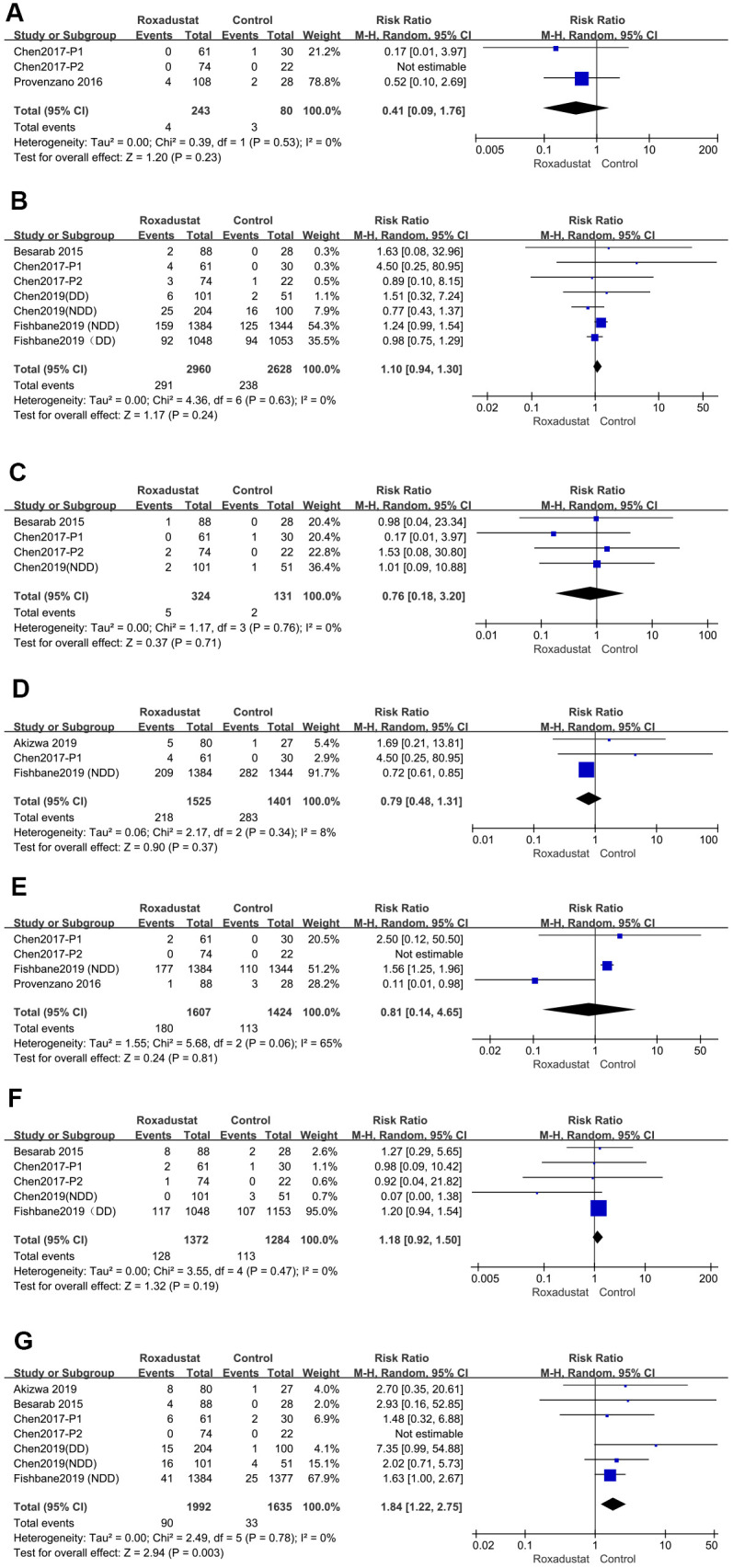
**Roxadustat effect on common AEs.** Forest plots of (**A**) cardiac-specific AE, (**B**) hypertension (**C**) liver injury (**D**) worsening chronic renal failure (in NDD subgroup), (**E**) urinary tract infections (UTI), (**F**) diarrhea, (**G**) hyperkalemia.

The most common AEs were cardiac-specific AE, hypertension, liver injury, worsening chronic renal failure (only in the NDD subgroup), urinary tract infections (UTIs), diarrhea, and hyperkalemia [14–22]. These data are presented as forest plots in Figure 8. There was no significant heterogeneity in the AEs except in the UTIs. There was no difference between the occurrence of AEs between the roxadustat and the placebo/ESA groups in terms of cardiac-specific AEs, hypertension, liver injury, worsening chronic renal failure (in the NDD subgroup), UTIs, or diarrhea. However, hyperkalemia was more common in the treatment group than in the placebo/ESA group (*P*=0.003, relative risk (RR)=1.84, 95% CI: 1.22, 2.75).

### Withdrawal comparison

**Figure 9:**
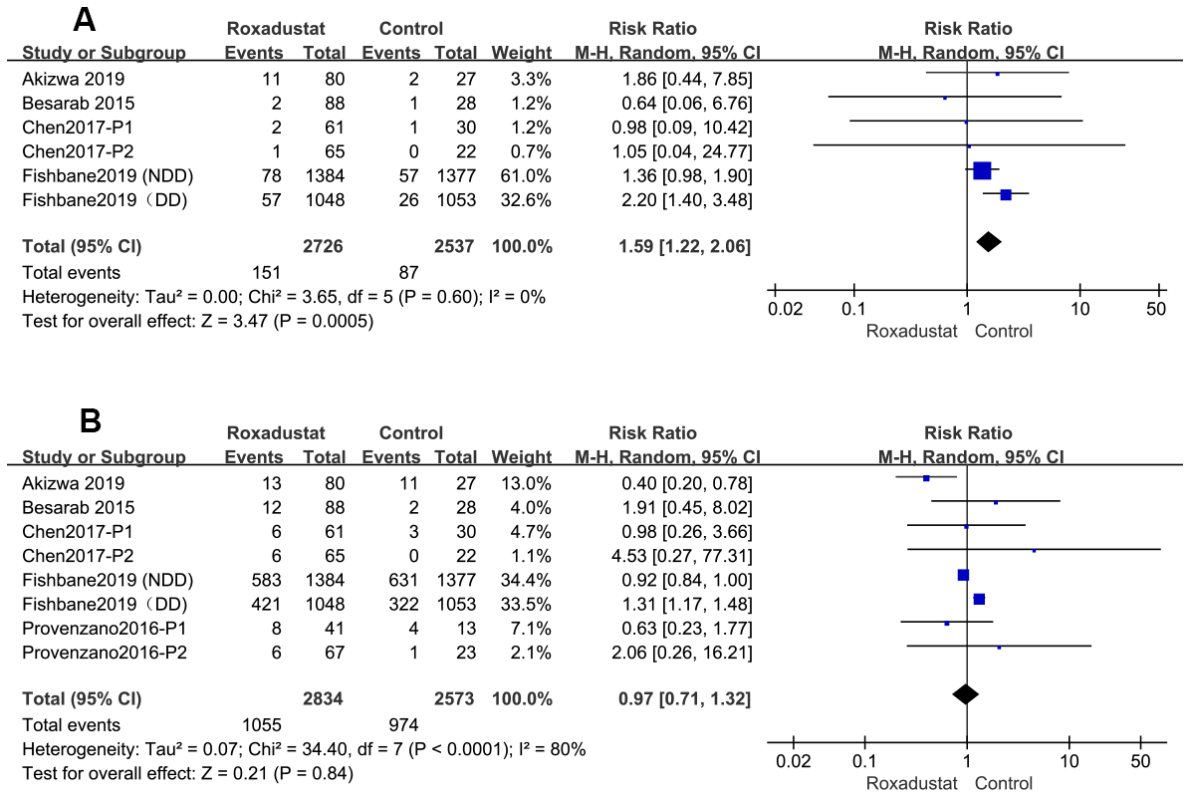
The rate of Roxadustat withdrawal because of AE (**A**) or any other reasons (**B**).

The rate of withdrawal from the study because of AEs was significantly higher in the roxadustat group than in the control group (P=0.0005, RR=1.59, 95% CI: 1.40, 2.06), without any significant heterogeneity (Figure 9A). However, there was no significant difference in the rate of the discontinuation of treatment by any cause between the two groups (Figure 9B).

## DISCUSSION

### Principal findings and relationship to other systematic reviews

As there were differences in the treatment of control groups in DD and NDD patients and the dosage groups of roxadustat, we performed subgroup analysis. This meta-analysis suggests that roxadustat significantly increases the Hb level compared to a placebo and has a higher Hb response rate than the placebo in the NDD subgroup. These findings are similar to the results of Zhong et al. meta-analysis [21] of HIF-PHIs. In the DD subgroup, there was no significant difference in the change in Hb levels from the baseline or the Hb response rate between the roxadustat and ESA groups. This observation supports the findings of Zhong et al [21]. Thus, roxadustat had a similar effect of elevating serum Hb levels as that observed for ESAs. Furthermore, there was no significant difference in mortality in CKD patients receiving roxadustat compared with the placebo/ESA group.

We found that roxadustat reduced serum hepcidin and ferritin levels more effectively in the NDD subgroup than in the DD subgroup. Hepcidin is a peptide that impairs iron absorption [13] and inhibits ferroportin from exporting iron from inside the cells. The level of hepcidin is increased by inflammation [7] and may induce resistance to ESAs. This effect of hepcidin on iron metabolism explains why an intravenous (IV) injection of iron results in high ferritin levels; reflecting the accumulation of iron in macrophages^15^. Serum ferritin is another biomarker of iron deficiency that can reflect the intracellular storage of iron. Intracellular iron forms a complex with cytoplasmic ferritin. Neither IV injection of iron nor red blood cell (RBC) transfusions were permitted until the end of the treatment in any of the trials in this meta-analysis. As most patients received oral iron except for those receiving rescue therapy, it is likely that the ferritin levels reflect the effects of the test drugs on iron storage. Therefore, we inferred that roxadustat helps iron absorption and promotes iron mobilization, and that its effect was similar to that of ESAs.

This analysis also showed that roxadustat significantly increased the TIBC levels in all the CKD patients compared to those in the placebo/ESA group. We found that TSAT levels decreased, and that the change from the baseline was significantly higher in the roxadustat group than in the placebo group in the NDD subgroup analysis. Thus, we hypothesize that roxadustat improves iron mobilization to prevent iron deficiency. This same observation was made by Del Vecchio et al. in their investigation of molidustat, another HIF-PHI [22]. However, the ΔTSAT of the roxadustat group was lower than that in the patients treated with ESAs in the DD subgroup. Hence, we speculate that the increased serum iron concentration in the roxadustat group was higher than that in the ESA-treated patients in terms of ΔTIBC. This hypothesis supports the effect of roxadustat on enteric iron absorption [17]. However, another explanation is that the DD patients relied on IV iron, without which the serum iron concentration would decrease in ESA-treated patients and become significantly lower than the serum iron concentration in the roxadustat subjects [23]. However, the change in the serum iron levels was seldom reported in the trials included in this meta-analysis. The serum iron levels could be strongly affected by the serum transferrin levels, which may be elevated by roxadustat treatment [17] and warrant further investigation.

Our data suggest that roxadustat does not increase the incidence of TEAEs and SAEs compared to the placebo or ESAs, which was consistent to the findings of Zhong et al [21]. We did not find any increase in the incidence of cardiac-specific AEs, hypertension, liver injury, chronic renal failure progression (only in NDD patients), UTI, or diarrhea. However, the incidence of hyperkalemia was higher in the roxadustat group than in the control group (*P*=0.003, RR=1.84, 95% CI: 0.82, 1.50). This is the first time that a meta-analysis of HIF-PHIs has identified an association between HIF-PHIs and hyperkalemia. Therefore, serum potassium concentrations may need to be closely monitored during the treatment of CKD patients with roxadustat. However, roxadustat has been shown to be well tolerated in phase II and III clinical trials [11].

### Implications for policymakers and clinicians

Roxadustat inhibits the degradation of HIFα, which dimerizes with HIFβ after accumulating in the cytoplasm. Following this, the dimer translocates into the nucleus and activates the transcriptional response to hypoxia to promote endogenous erythropoiesis [10]. Because roxadustat adjusts the Hb and iron level via a mechanism that is different from that of ESAs, it may replace ESAs in the treatment of anemia in CKD patients [22]. Our meta-analysis suggests that roxadustat is a safe and effective drug for the treatment of anemia in CKD. Like ESA treatment, roxadustat increases serum Hb levels, although it may induce hyperkalemia as a potential side effect. There are many publications demonstrating that HIF is an iron sensor [24], and that HIF-PHIs could improve intestinal iron absorption by suppressing hepcidin expression and increasing the expression of iron transport enzymes that could deliver iron into the bone marrow. These effects of HIF-PHIs could increase the efficacy of oral iron therapy [17] in anemia and reduce the risk of allergic reactions and infection associated with IV iron therapy.

Moreover, Sakaguchi et al [8] reported that patients receiving long-acting ESA treatment had a higher mortality rate than those treated with short-acting ESAs. Whether the long-term use of HIF-PHIs has a similar effect and whether it affects the number of cardiovascular events or the risk of cancer development is not yet known [6, 8]. Furthermore, whether the stimulation of the production of endogenous EPO by HIF-PHIs will last for the long term in CKD patients is unknown [25]. These questions may be answered by ongoing clinical trials that will be completed over the next several years [26, 27] and influence the future applications of roxadustat in CKD.

There are several other HIF-PHIs currently in clinical trials, such as molidustat, daprodustat, vadadustat, enarodustat, and DS-1093a, which inhibit different PHD enzymes and have different half-lives. Most of these inhibitors stimulate endogenous EPO expression from the kidney and liver [27]. Recently, Sota Kato et al. reported a novel HIF-PHI, TP0463518, that could stabilize HIF-2α and induce EPO production specifically from the liver [28]. Although TP0463518 may have some advantages, more clinical trials are required to determine if it is as efficacious and safe as roxadustat.

To conclude, roxadustat is a promising drug for the treatment of CKD-induced anemia; and it may have several advantages over traditional ESAs: ① it is orally active and is more effective in NDD-CKD and PD patients; ②it suppresses hepcidin production more effectively; and ③ it may result in increased efficacy of oral iron therapy and reduce the requirement of IV iron [29].

### Strengths and weaknesses of the review

This meta-analysis may be the first to present the analysis of results for only roxadustat and characterize the common AEs associated with roxadustat treatment. However, our study has some limitations. First, the number of included RCTs was <10, and we could not use a funnel figure to analyze the publication bias. Second, some critical results could not be presented by the articles we included. We planned to analyze the blood pressure, serum cholesterol, and platelet counts in the treatment and control groups to determine the cardiovascular safety of roxadustat. However, these data were not examined or presented in sufficient detail in the RCTs included in this meta-analysis. Finally, the included RCTs were only conducted over short periods for treatment and follow-up because the drug is relatively new, clinical trials were started not long ago. Moreover, the dosage strategies of the drugs varied in all the studies. Thus, these aspects of the RCTs could contribute to the heterogeneity in our results. Therefore, we need to include more long-term and high-quality trials to investigate the long-term efficacy and safety of roxadustat in CKD patients in the future.

## CONCLUSIONS

Our meta-analysis showed that roxadustat is a safe and effective drug for treating anemia in CKD patients. It had a similar effect of serum Hb elevation as that observed for ESAs. Roxadustat raises the Hb level more significantly and has a higher Hb response rate in NDD patients than the placebo group. It may induce hyperkalemia, although this may be well tolerated in CKD patients.

## MATERIALS AND METHODS

This meta-analysis was conducted according to the guidelines of the Cochrane Handbook for Systematic Reviews of Interventions [30].

### Search strategy

We searched EMBASE, PubMed, MEDLINE, Cochrane Database, and Google Scholar from their inception up to October 31, 2019, without any language limitations. We searched the database by using the Medical Subject Headings (MeSH) terms and the corresponding keywords. The keywords used for all searches were "roxadustat", "hypoxia-inducible factor*", "HIF*", "prolyl hydroxylase* inhibitor", "HIF-PH*", "prolyl hydroxylase* inhibitor hypoxia-inducible factor*", "FG-4592*" and "anemia", "anemia", "hypohemia", "Spanemia" and "CKD", "Chronic kidney disease*", "Renal Insufficiency*", "Kidney Insufficiency*", and "Renal disease*". https://clinicaltrials.gov/ was also searched and we manually identified other potentially appropriate trials by checking the bibliographies of the included trials and previous reviews.

### Inclusion and exclusion criteria

### Non dialysis-dependent (NDD) study

Inclusion criteria: ① 18 to 80 years old CKD patients with an eGFR using the modification of diet in renal disease of ≤89 mL/min/1.73 m^2^; ② the patient does not require dialysis; ③ a baseline Hb of <10.0 g/dL; ④RCT.

Exclusion criteria: ① Any history of thromboembolic events; ② patients with severe hypertension [diastolic blood pressure (BP) > 109 mmHg or systolic BP > 170 mmHg at screening]; ③ a history of treatment with ESA injection or RBC transfusion within the previous six weeks; ④ patients with causes of anemia other than CKD.

### DD study

Inclusion criteria: ① 18 to 80 years old and receiving maintenance hemodialysis (HD) or peritoneal dialysis (PD); ② a mean Hb level between 9.0 and 12.0 g/dL Hb in three screening tests; ③ has received stable doses of EA during the previous seven weeks; ④ RCT. Exclusion criteria: ① a recent history of cardiovascular events; ② patients with causes of anemia other than CKD.

### Data extraction and risk of bias assessment

Li Zhang assessed the search results according to their relevance to the present study and removed the irrelevant records. The titles and abstracts of the remaining records were then assessed for their relevance to the inclusion criteria by two independent reviewers (Li Zhang and Shuai Xue). Any disagreement was resolved through discussion between the two reviewers or by consulting a third reviewer. Li Zhang assessed the risk of bias of each included study using the relevant, validated tool for each study design. Jia Li checked the risk of bias in each assessment. The risk of bias in these studies was assessed using the assessment tool of the Cochrane RCTs risk bias.

### Statistical analysis

We used the Review Manager (RevMan) 5.3 software (Nordic Cochrane Centre) to conduct the meta-analysis. We used relative risks with 95% confidence intervals for dichotomous data and MDs with 95% CIs were calculated for continuous data. The heterogeneity across the studies was assessed using a Cochran Q test, data were considered statistically significant when the I^2^ statistic P-value was less than 0.1 and I^2^ was over 50% [31]. To account for clinical heterogeneity, we used a random-effects model and a subgroup analysis depending on whether the patients were DD or NDD. We could not assess the publication bias using the funnel figure because the number of analyzed trials was less than ten. Furthermore, we conducted a sensitivity analysis by excluding one study at a time to test its influence on the outcomes of the analysis.

### Availability of data and material

All relevant data are within the manuscript and its Supporting Information files.

### Supporting information

Supplementary Table 1, Baseline characteristics of included studies.

Supplementary Table 2, PRISMA checklist.

Supplementary Table 3, Search strategy for PubMed.

## Supplementary Material

Supplementary Table 1

Supplementary Table 2

Supplementary Table 3
