# Supplementary material for: Roxadustat for the treatment of anemia in patients with chronic kidney diseases: a meta-analysis
Source: Aging (Albany NY). 2021 Jun 11;13(13):17914–29. doi: 10.18632/aging.203143 (PMC8312415; doi:10.18632/aging.203143)
Supplement: Supplementary Table 1 [file aging-13-203143-s001.doc]

**Supplementary Table 1. Baseline characteristics of included studies.**

| Study | Country | Patients | Control | Duration (weeks) | Age | Sex,  Male | eGFR (mL/mil/1.73m^2^) | Hb(g/dL) | TSAT% | Ferritin  (ng/mL) | Hepcidin  (ng/mL) | Trial  Phase | Clinical Study type | NCT# |
| --- | --- | --- | --- | --- | --- | --- | --- | --- | --- | --- | --- | --- | --- | --- |
| Besarab2015^[15]^ | USA | NDD-CKD | Placebo | 40 | I:64.0 | I:33 | I:34.3 | I:10.3 | I:28.4 | I: 199 | N/A | 2 | Multicenter,randomized,placebo-controlled | NCT00761657 |
|  |  |  |  |  | C:65.8 | C:49 | C:33.6 | C:10.3 | C:28.4 | C:228 |  |  |  |  |
| Provenzano2016^[16]^ | USA | DD-CKD | EA | 14  (Part1） | I: 55.8 | I:66 | N/A | I:11.3 | I:30.4 | I:912.5 | N/A | 2 | Randomized,6-to19-Week,Open-Label | NCT02273726 |
|  |  |  |  |  | C:59.5 | C:69 |  | C:11.5 | C:30.4 | C:875.6 |  |  |  |  |
|  |  |  |  | 23  (Part2） | I:56.9 | I:67 | N/A | I:11.2 | I:29.2 | I:827.7 | N/A |  |  |  |
|  |  |  |  |  | C:57.0 | C:61 |  | C:11.2 | C:28.1 | C:1,065.8 |  |  |  |  |
| Chen2017-P1^[17]^ | China | NDD-CKD | Placebo | 8 | I:49.7 | I:28.6 | I:20.5 | I:8.8 | I:22.7 | I:202 | I:71.5 | 2 | Double-blinded NDD study | NCT01599507 |
|  |  |  |  |  | C:51.4 | C:26.7 | C:23.0 | C:8.9 | C:21.9 | C:221 | C:69.9 |  |  |  |
| Chen2017-P2^[17]^ | China | DD-CKD | EA | 6 | I:50.8 | I:60.4 | N/A | I:10.7 | I:32.2 | I:453 | I:182.9 | 2 | Open-label study | NCT01596855 |
|  |  |  |  |  | C:53.8 | C:59.1 |  | C:10.6 | C:34.1 | C:458 | C:209.0 |  |  |  |
| Akizwa 2019^[18]^ | Japan | NDD-CKD | Placebo | 24 | I:64.4 | I:48.8 | I:16.3 | I:9.4 | I:29.7 | I:131.1 | I:39.9 | 2 | Randomized,Double-Blind,Placebo-Controlled | NCT01964196 |
|  |  |  |  |  | C:61.9 | C:40.7 | C:16.3 | C:9.3 | C:26.8 | C:125.4 | C:40.9 |  |  |  |
| Chen2019(NDD)^[19]^ | China | NDD-CKD | Placebo | 26 | I:54.7 | I:36 | I:16.5 | I:8.9 | I:20.6 | I:191.4 | N/A | 3 | Initial 8 week double blind placebo- controlled phase+18week open- label phase | NCT02652819. |
|  |  |  |  |  | C:53.2 | C:39 | C:14.5 | C:8.9 | C:23.0 | C:266.2 |  |  |  |  |
| Chen2019(DD)^[20]^ | China | DD-CKD | EA | 26 | I:47.6 | I:61.8 | N/A | I:10.4 | I:33.8 | I:498.5 | N/A | 3 | Randomized,open-label,active-controlled | NCT02652806 |
|  |  |  |  |  | C:51.0 | C:58 |  | C:10.5 | C:30.0 | C:420.1 |  |  |  |  |
| Fishbane2019(NDD)^[21]^ | US and EU | NDD-CKD | Placebo | 52 | I:60.9 | I:40.8 | I:19.7 | I:9.1 | N/A | N/A | N/A | 3 | Randomized,Double-Blind,Placebo-Controlled,International Study | N/A |
|  |  |  |  |  | C:62.4 | C:43.8 | C:20.0 | C:9.1 |  |  |  |  |  |  |
| Fishbane2019(DD)^[22]^ | US and EU | DD-CKD | ESAs | 52 | I:53.5 | I:59.5 | N/A | I:9.99 | N/A | N/A | N/A | 3 | International,Randomized,Open-Label, Active-Controlled Study | N/A |
|  |  |  |  |  | C:54.5 | C:59.3 |  | C:10.02 |  |  |  |  |  |  |

**Abbreviations**:N/A:Not applicable； I:Intervening group;C:Control group;EA:Epoetin alfa;ESA:erythropoiesis-stimulating agents;SD:standard deviation
