# Supplementary material for: Roxadustat for the treatment of anemia in patients with chronic kidney diseases: a meta-analysis
Source: Aging (Albany NY). 2021 Jun 11;13(13):17914–29. doi: 10.18632/aging.203143 (PMC8312415; doi:10.18632/aging.203143)
Supplement: Supplementary Table 3 [file aging-13-203143-s003.pdf]

SUPPLEMENTARY TABLE

Supplementary Table 3. Search strategy for PubMed.

**Recent queries in pubmed**			
**Search**	**Query**	**Items found**	**Time**
#23	Search (#21 AND #22)	38	4:25:10
#22	Search ((randomized controlled trial[pt] OR controlled clinical trial[pt] OR randomized[tiab] OR placebo[tiab] OR clinical trials as topic[mesh:noexp] OR randomly[tiab] OR trial[ti]) NOT (animals [mh] NOT (humans [mh] AND animals[mh])))	1152604	4:24:41
#21	Search (#15 AND #20)	154	4:22:28
#20	Search (#16 OR #17 OR #18 OR #19)	122784	3:56:50
#19	Search Renal disease*[Title/Abstract]	63282	3:56:04
#18	Search Kidney Insufficienc*[Title/Abstract]	683	3:55:42
#17	Search Renal Insufficienc*[Title/Abstract]	22549	3:55:16
#16	Search (CKD[Title/Abstract] OR Chronic kidney disease*[Title/Abstract])	48966	3:54:52
#15	Search (#8 AND #14)	498	3:50:03
#14	Search (#9 OR #10 OR #11 OR #12 OR #13)	218603	3:49:33
#13	Search spanemia[Title/Abstract] Schema: all	0	3:48:44
#12	Search spanemia[Title/Abstract]	0	3:48:44
#11	Search Hypohemia[Title/Abstract]	1	3:46:02
#10	Search (anemia[Title/Abstract] OR anaemia[Title/Abstract])	143529	3:45:31
#9	Search anemia[mesh]	157673	3:44:25
#8	Search (#1 OR #2 OR #3 OR #4 OR #5 OR #6 OR #7)	28202	3:42:54
#7	Search hypoxia-inducible factor*[Title/Abstract]	16919	3:40:31
#6	Search HIF*[Title/Abstract]	25328	3:40:16
#5	Search prolyl hydroxylase* inhibitor[Title/Abstract]	488	3:40:01
#4	Search hypoxia-inducible factor* prolyl hydroxylase* inhibitor[Title/Abstract]	370	3:39:38
#3	Search HIF-PH*[Title/Abstract]	71	3:38:57
#2	Search FG-4592*[Title/Abstract]	37	3:38:34
#1	Search Roxadustat*[Title/Abstract]	39	3:38:02
